# Violent suicide methods across life stages - a national population-based register study

**DOI:** 10.3389/fpsyt.2025.1715801

**Published:** 2026-01-20

**Authors:** Henrik Imberg, Maja Magdalena Olsson, Joakim Öhlén, Cecilia Larsdotter, Margda Waern, Christopher Holmberg

**Affiliations:** 1 Statistiska Konsultgruppen, Gothenburg, Sweden; 2 Department of Molecular and Clinical Medicine, Institute of Medicine, Sahlgrenska Academy, University of Gothenburg, Gothenburg, Sweden; 3 Department of Nursing Science, Sophiahemmet University, Stockholm, Sweden; 4 Institute of Health and Care Sciences, Sahlgrenska Academy, University of Gothenburg, Gothenburg, Sweden; 5 Centre for Person-centred Care (GPCC), University of Gothenburg, Gothenburg, Sweden; 6 Palliative Centre, Sahlgrenska University Hospital, Gothenburg, Sweden; 7 Institute of Neuroscience and Physiology, Sahlgrenska Academy, University of Gothenburg, Gothenburg, Sweden; 8 Department of Psychotic Disorders, Sahlgrenska University Hospital, Gothenburg, Sweden

**Keywords:** age factors, life cycle stages, logistic models, suicide methods, thanatology

## Abstract

**Aim:**

To examine the use of violent suicide methods across life stages, and associations with sociodemographic characteristics, healthcare utilization, and place of death.

**Methods:**

Data from Swedish national registers encompassing all recorded suicide deaths between 2013 and 2019 were analyzed. Suicide methods were categorized as violent or non-violent. Associations were assessed using univariable and multivariable logistic regression, with age modeled using restricted cubic splines to capture nonlinear effects. Interaction analyses were conducted to examine how associations varied by age.

**Results:**

A total of 8,325 suicides were included, with 77% involving violent methods. The likelihood of violent method use peaked at age 20 and declined until around age 60, after which a modest increase was observed. The use of violent suicide methods was stable across all ages for men but there was a steady decrease in women until age 60. Foreign-born older adults were less likely to use violent methods than their Swedish-born counterparts. Lower educational attainment, living with others, and dying in nursing homes or other institutional settings were also associated with higher odds of violent method use.

**Conclusions:**

This study contributes to the literature by highlighting distinct age- and subgroup-specific trends in the use of violent suicide methods. We noted a consistent decrease among women up to age 60, and lower rates among older migrants. These insights underscore the value of a lifespan approach in understanding suicide risk and method choice, and point to the need for tailored, context-aware prevention strategies.

## Introduction

Suicide is a major global public health challenge claiming the lives of over 700,000 individuals each year, with countless more attempting suicide (1). It affects people of all ages, genders, and backgrounds, and is widely recognized as a complex phenomenon with profound personal and societal implications. Across Europe, suicide is a leading cause of years of life lost, particularly among young people (2). In Sweden, 1,226 individuals died by suicide in 2021, with men accounting for 71% and women 29% (3). Among people aged 15 and over, Sweden’s suicide rate in 2023 was 15.2 per 100,000. Rates were highest in men aged ≥85 and lowest in women aged 15–29. Among those under 19, girls and boys die by suicide in similar numbers (3).

A key component of suicide prevention is understanding the methods individuals use. Violent methods (defined as traumatic, rapid-acting methods such as firearms), when used in non-fatal attempts, are generally associated with a markedly increased risk of subsequent suicide (4). The percentage of case fatalities differs markedly between methods, from 75–90% for violent methods to 8% for less violent methods, such as drug poisoning (5). This has prevention implications because persons who use more violent methods are less likely to have a non-lethal prior attempt that would serve as a flag for treatment (6).

Emerging research underscores the importance of understanding how suicide methods vary across life stages. As certain risk factors seem to be more salient in younger versus older age groups, this calls for tailored prevention strategies (7). Adolescents and young adults, for instance, may act more impulsively, with suicides often occurring after a crisis, while late-life suicides may occur in the context of chronic illness, serious health issues, social isolation, or bereavement (8). Despite the known differences in age-related risk factors, there is limited understanding of how this relates to method choice, and particularly how this varies with age. Understanding these patterns is essential, as adverse events and risk exposures accumulate differently across the lifespan. Sex-based differences also warrant attention, with men and women exhibiting distinct patterns in method selection and healthcare engagement (9). For example, Hempstead et al. (10) showed that firearms are disproportionately used in male suicides, particularly when physical health is listed as a contributing circumstance.

Contextual factors, such as location of death, are also relevant, as method use is often shaped by environmental aspects (e.g. suicides by vehicle collision). The availability of specific methods in particular settings may act as a catalyst, converting suicidal ideation to action (11). In Sweden, the estimated rate of civilian firearms per 100 residents is 23.1 compared with 120.5 in the United States (12). Gun ownership is regulated in Sweden and physicians must report to the police when they deem that a patient should not have gun access for medical (most commonly psychiatric) reasons. However, a recent joint report from the Swedish National Board of Health and Welfare and the Swedish Police Authority (13) indicates that this reporting system requires improvement, particularly in primary care, underscoring how method availability and regulatory frameworks shape suicide risk and must be considered when examining method choice. Together, these patterns underline the importance of incorporating not only individual characteristics but also societal and environmental contexts into research on suicide across the life course (14).

We sought to examine the use of violent suicide methods across life stages, including sex interactions, and to investigate their associations with sociodemographic characteristics, healthcare utilization, and place of death.

## Methods

### Data sources

Data for this study were obtained from the Swedish Cause of Death Register at the National Board of Health and Welfare (NBHW), which includes all individuals in Sweden who died between 2013 and 2019. The register is validated for accuracy (15). Study inclusion criteria included all deaths with an underlying cause of death classified as intentional self-harm. Data were retrieved from Statistics Sweden (microdata online access – MONA), and the NBHW (patient data register). For research reporting, Strengthening the Reporting of Observational Studies in Epidemiology (STROBE) Statement: Guidelines for Reporting Observational Studies was followed (16). See **Supplementary Table 1** for the checklist.

### Study variables

The ICD codes X60–X82 were used to capture the deaths following intentional self-harm. We excluded 53 cases (0.6%) with ICD-10 codes indicating unspecified or other means (X83, X84) and sequelae of self-harm (Y87.0) since method violence could not be determined.

There is no consensus regarding the definition of violent vs. non-violent suicide methods (17). Our categorization was similar to that of Stenbacka and Jokinen (18). In this context, “violent” and “non-violent” refer to the mode of injury (traumatic vs non-traumatic) rather than to intent or moral connotations. We emphasize that method type cannot be equated simply with degree of planning or severity. Several non-traumatic serious suicide attempts (e.g., using potent medications such as insulin or warfarin) can be highly planned, as illustrated by prior work (19). The full classification is presented in **Table 1**.

**Table 1:** ICD-10-codes, definitions, and classification of suicide methods as non-violent and violent.

ICD-10 code and definition
*Non-violent methods*
X60: Intentional self-poisoning by and exposure to nonopioid analgesics, antipyretics and antirheumatics
X61: Intentional self-poisoning by and exposure to antiepileptic, sedative, hypnotic, anti-Parkinson, and psychotropic drugs, not elsewhere classified
X62: Intentional self-poisoning by and exposure to narcotics and psychodysleptics [hallucinogens], not elsewhere classified
X63: Intentional self-poisoning by and exposure to other drugs acting on the autonomic nervous system
X64: Intentional self-poisoning by and exposure to other and unspecified drugs, medicaments and biological substances
X65: Intentional self-poisoning by and exposure to alcohol
X67: Intentional self-poisoning by and exposure to other gases and vapors
X67.8: Intentional self-poisoning by and exposure to other gases and vapors - at other specified places
X69: Intentional self-poisoning by and exposure to other and unspecified chemicals and noxious substances
*Violent methods*
X66: Intentional self-poisoning by and exposure to organic solvents and halogenated hydrocarbons and their vapors
X68: Intentional self-poisoning by and exposure to pesticides
X70: Intentional self-harm by hanging strangulation and suffocation
X71: Intentional self-harm by drowning and submersion
X72: Intentional self-harm by handgun discharge
X73: Intentional self-harm by rifle, shotgun and larger firearm discharge
X74: Intentional self-harm by other and unspecified firearm discharge
X75. Intentional self-harm by explosive material
X76. Intentional self-harm by smoke, fire and flames
X78. Intentional self-harm by sharp object
X80: Intentional self-harm by jumping from a high place
X81. Intentional self-harm by jumping or lying before moving object
X82: Intentional self-harm by crashing of motor vehicle

Individual-level variables included the following: demographic, geographic, healthcare utilization, and socioeconomic characteristics, sex (female vs. male), age (in years), region of birth (Sweden vs. other countries), marital status (never married, married/registered partner, widowed, divorced), living with others (yes/no, i.e., household composition including cohabiting partners as well as other adult household members such as adult children), presence of children under 18 years in the household (yes vs. no), and educational level (less than mandatory, secondary, or higher). Place of death was categorized as hospital, home, nursing home (i.e. a residential facility offering 24-hour assistance with daily activities, health care, rehabilitation, or palliative and hospice care) (20), or other locations (e.g. public spaces, workplaces). Healthcare utilization included emergency department (ED) visits and hospital admissions the month before death. Geographic classification, as defined by Statistics Sweden (SCB), differentiates between urban and rural areas. Urban areas are defined as having a population of at least 200 inhabitants and building density not exceeding 200 meters between buildings. The variable ‘healthcare region’ reflects large administrative health-care areas that differ in population density, urban/rural mix, and service organization. They were grouped into six national clusters: Uppsala-Örebro North, Stockholm, West, Southeast, and South. The regions include major urban centers (such as Stockholm and West) which have the highest population density and broad access to specialized care, whereas some other regions (e.g. North) have more dispersed populations and generally greater travel distances to services.

### Statistical analysis

Descriptive data were summarized as counts and percentages for categorical variables. As the proportion of missing data was below 5%, no imputation was performed, and a complete-case analysis was applied (21).

Associations between suicide method (violent vs. non-violent) and covariates were assessed using univariable and multivariable logistic regression models. All variables were included in the multivariable analyses. Results are reported as odds ratios (ORs) with 95% confidence intervals (CIs). Age was modelled as a continuous variable using restricted cubic splines with knots at the 10th, 50th, and 90th percentiles to capture potential nonlinear effects (22). ORs were calculated for ages 20, 30, 40, 50, 70, and 80 years, using age 60 as the reference. Interaction analyses were conducted to evaluate whether the associations between violent suicide methods and covariates varied by age. In addition, sex-stratified logistic regression models and sex interaction analyses were performed to examine sex-specific patterns in the use of violent methods.

To provide a more detailed description of method choice, we also performed a *post-hoc* analysis categorizing suicide methods into four groups: self-poisoning (ICD-10 X60–X69), hanging/suffocation (X70), firearms (X72–X74), and other methods (X71 and X75–X82). These distributions were examined overall and by age and sex.

All statistical analyses were conducted at the 5% significance level using SAS/STAT^®^ Software, version 9.4 (SAS Institute Inc., Cary, NC, USA).

## Results

### Characteristics of the study population

Between 2013 to 2019, a total of 599,171 deaths were recorded with a registered place of death. Among these, 8,325 were classified with an ICD-10 code indicating intentional self-harm as the underlying cause. The proportion of violent suicide deaths remained stable over this time period (**Supplementary Figure 1**). Descriptive characteristics of the study population are presented in **Table 2**. The mean age of death was 50.7 years (SD: 19.7). Most individuals who died by suicide were male (70%) and born in Sweden (86%). Over half had completed upper secondary education (53%), were unmarried (51%), or lived with others (56%). The majority (84%) resided in urban areas. More than half (58%) died at home, while for 30% the place of death was reported as ‘other/unknown’, precluding further classification. Additionally, 19% of individuals had visited an emergency department in the month preceding their death, and a similar proportion had at least one hospital admission during their final month of life (**Table 2**).

**Table 2:** Distribution of sociodemographic characteristics, place of death, and healthcare utilization prior to death among 8,325 suicides in Sweden, 2013 and 2019.

Characteristic	Mean (SD) / n (%)
Age at death, mean (SD)	50.7 (19.7)
Male sex	5,818 (70%)
Born in Sweden	7,151 (86%)
Educational attainment ^a^	
Less than mandatory education (<9 years)	833 (10%)
Mandatory education (9 years)	1,468 (18%)
Secondary education (12 years)	4,362 (53%)
Higher education (>12 years)	1,497 (18%)
Marital status ^b^	
Married	1,839 (22%)
Never married	4,262 (51%)
Widowed	582 (7%)
Divorced	1,612 (19%)
Living alone	3,612 (44%)
Children (<18 years) in household	1,377 (17%)
Residing in an urban area	6,963 (84%)
Healthcare region ^c^	
Uppsala-Örebro	1,854 (22%)
North	796 (10%)
Stockholm	1,660 (20%)
West	1,665 (20%)
Southeast	864 (10%)
South	1,456 (18%)
Place of death	
Hospital	802 (10%)
Nursing home	150 (2%)
Home	4,834 (58%)
Other/Unknown	2,539 (30%)
Emergency department visit month before death	1,583 (19%)
Hospital admission month before death	1,545 (19%)

a 165 observations missing.

b 30 observations missing.

c 30 observations missing.

### Distribution of violent suicide methods

The most common suicide methods were hanging, strangulation, and suffocation, accounting for 45% of all cases. Self-poisoning by pharmaceuticals, unspecified drugs, or other drugs was the second most common method (21%), followed by firearms and explosives (9%). Women most often used self-poisoning (39%) and had a higher proportion of drowning deaths (8%), whereas men predominantly used violent methods, particularly hanging (49%) and firearms (13%). Further details are provided in **Table 3**.

**Table 3:** Distribution of suicide methods in the full cohort and by sex.

Method	Full cohort (n=8,325)	Female (n=2,507)	Male (n=5,818)
Self-poisoning ^a^	1,764 (21.2%)	973 (38.8%)	791 (13.6%)
Gases, organic solvents, vapors ^a^	120 (1.4%)	14 (0.6%)	106 (1.8%)
Hanging, strangulation, suffocation	3,708 (44.5%)	866 (34.5%)	2,842 (48.8%)
Drowning and submersion	403 (4.9%)	202 (8.1%)	201 (3.5%)
Firearms and explosives	771 (9.3%)	17 (0.7%)	754 (13.0%)
Smoke, fire and flames	125 (1.5%)	34 (1.4%)	91 (1.6%)
Cutting or piercing	268 (3.2%)	43 (1.7%)	225 (3.9%)
Jumping from a high place	422 (5.1%)	137 (5.5%)	285 (4.9%)
Jumping/lying before moving object	592 (7.1%)	198 (7.9%)	394 (6.8%)
Crashing of motor vehicle	99 (1.2%)	12 (0.5%)	87 (1.5%)
Unspecified/other means	53 (0.6%)	11 (0.4%)	42 (0.7%)

a Non-violent methods; all others are classified as violent.

### Variations in violent suicide methods across life stages

**Figure 1:**
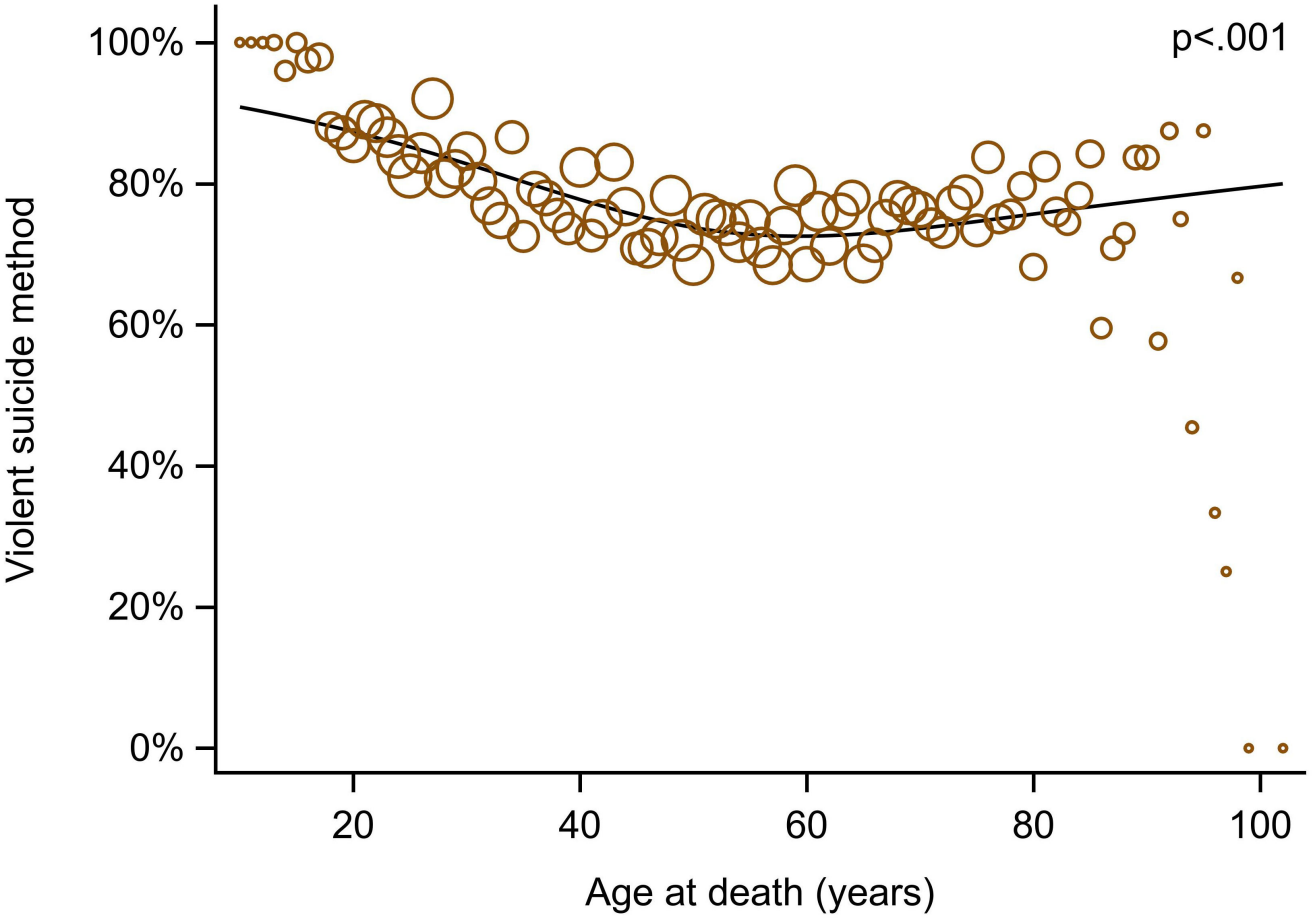
Association between age at death and use of violent suicide methods. Circles represent observed proportions by age, with size proportional to the number of observations. The line shows the fitted mean trend estimated using logistic regression with age modelled using restricted cubic splines.

There was a statistically significant association between age and the use of violent suicide methods, with higher rates observed at both younger and older ages (**Figure 1**). In multivariable analyses, the adjusted odds of using violent methods were higher at age 20 (OR 2.44, 95% CI 1.93–3.07) compared with age 60, decreased to a modest elevation at age 40 (OR 1.27, 95% CI 1.17–1.39), and showed no difference at age 50. The use of violent methods then increased again after age 60, with ORs of 1.09 (*p* = .020) at age 70 and 1.25 (*p* = .005) at age 80. Other factors independently associated with a higher likelihood of violent suicide methods included male sex (*p* <.001), lower educational attainment (*p* = .036), being married versus widowed (*p* = .002), living with others (*p* <.001), rural residence (*p* <.001), and residence in a nursing home (*p* = .003) (**Table 4**).

**Table 4:** Factors associated with the use of violent suicide methods.

Explanatory variable	Comparison	Univariable OR (95% CI)	*P*	Multivariable OR (95% CI)	*P*
Age	20 vs 60 years	2.62 (2.19, 3.13)	<.001	2.44 (1.93, 3.07)	<.001
	30 vs 60 years	1.82 (1.63, 2.04)	<.001	1.73 (1.49, 2.01)	<.001
	40 vs 60 years	1.32 (1.25, 1.40)	<.001	1.27 (1.17, 1.39)	<.001
	50 vs 60 years	1.06 (1.03, 1.10)	<.001	1.04 (0.99, 1.09)	0.092
	70 vs 60 years	1.06 (1.00, 1.11)	0.034	1.09 (1.01, 1.17)	0.020
	80 vs 60 years	1.18 (1.05, 1.32)	0.005	1.25 (1.07, 1.46)	0.005
Female		0.28 (0.25, 0.31)	<.001	0.29 (0.25, 0.32)	<.001
Born in Sweden		0.91 (0.79, 1.05)	0.20	1.06 (0.90, 1.25)	0.50
Educational attainment	Mandatory education vs Less than mandatory	0.70 (0.57, 0.87)	0.001	0.60 (0.47, 0.78)	<.001
	Secondary vs Less than mandatory	0.81 (0.67, 0.98)	0.028	0.79 (0.63, 0.98)	0.036
	Higher education vs Less than mandatory	0.74 (0.60, 0.92)	0.006	0.88 (0.69, 1.13)	0.33
Marital status	Never married vs Married	0.85 (0.74, 0.98)	0.029	0.86 (0.71, 1.04)	0.13
	Widowed vs Married	0.44 (0.35, 0.54)	<.001	0.65 (0.50, 0.85)	0.002
	Divorced vs Married	0.42 (0.36, 0.49)	<.001	0.60 (0.50, 0.73)	<.001
Living with others		1.76 (1.58, 1.95)	<.001	1.31 (1.14, 1.51)	<.001
Children under 18 years in household		1.46 (1.26, 1.70)	<.001	0.99 (0.82, 1.19)	0.90
Residing in urban area		0.57 (0.48, 0.67)	<.001	0.64 (0.54, 0.77)	<.001
Healthcare region	North vs Uppsala-Örebro	1.04 (0.84, 1.28)	0.73	1.05 (0.84, 1.32)	0.65
	Stockholm vs Uppsala-Örebro	0.74 (0.64, 0.87)	<.001	0.83 (0.70, 0.99)	0.039
	West vs Uppsala-Örebro	0.80 (0.68, 0.94)	0.007	0.86 (0.72, 1.03)	0.10
	Southeast vs Uppsala-Örebro	0.90 (0.74, 1.10)	0.31	0.94 (0.76, 1.17)	0.58
	South vs Uppsala-Örebro	0.89 (0.75, 1.05)	0.16	1.05 (0.87, 1.26)	0.61
Place of death	Nursing home vs Hospital	2.13 (1.34, 3.38)	0.001	2.17 (1.30, 3.62)	0.003
	Home vs Hospital	1.03 (0.87, 1.21)	0.76	1.01 (0.82, 1.24)	0.91
	Other place or unknown vs Hospital	4.35 (3.54, 5.34)	<.001	3.94 (3.10, 5.00)	<.001
ED visits one month prior to death		0.99 (0.87, 1.12)	0.84	1.19 (1.00, 1.42)	0.054
Hospital admission one month prior to death		0.82 (0.72, 0.93)	0.002	0.90 (0.75, 1.08)	0.25
Year of death		1.00 (0.98, 1.03)	0.83	1.00 (0.97, 1.03)	0.85

Statistical analyses were performed using logistic regression, with age modelled using restricted cubic splines.

All listed variables were included in the multivariable analyses.

Causes of death coded as X83, X84 and Y87.0 (unspecified or sequalae of self-harm) were excluded from the analysis.

CI, confidence interval; ED, emergency department.

**Figure 2:**
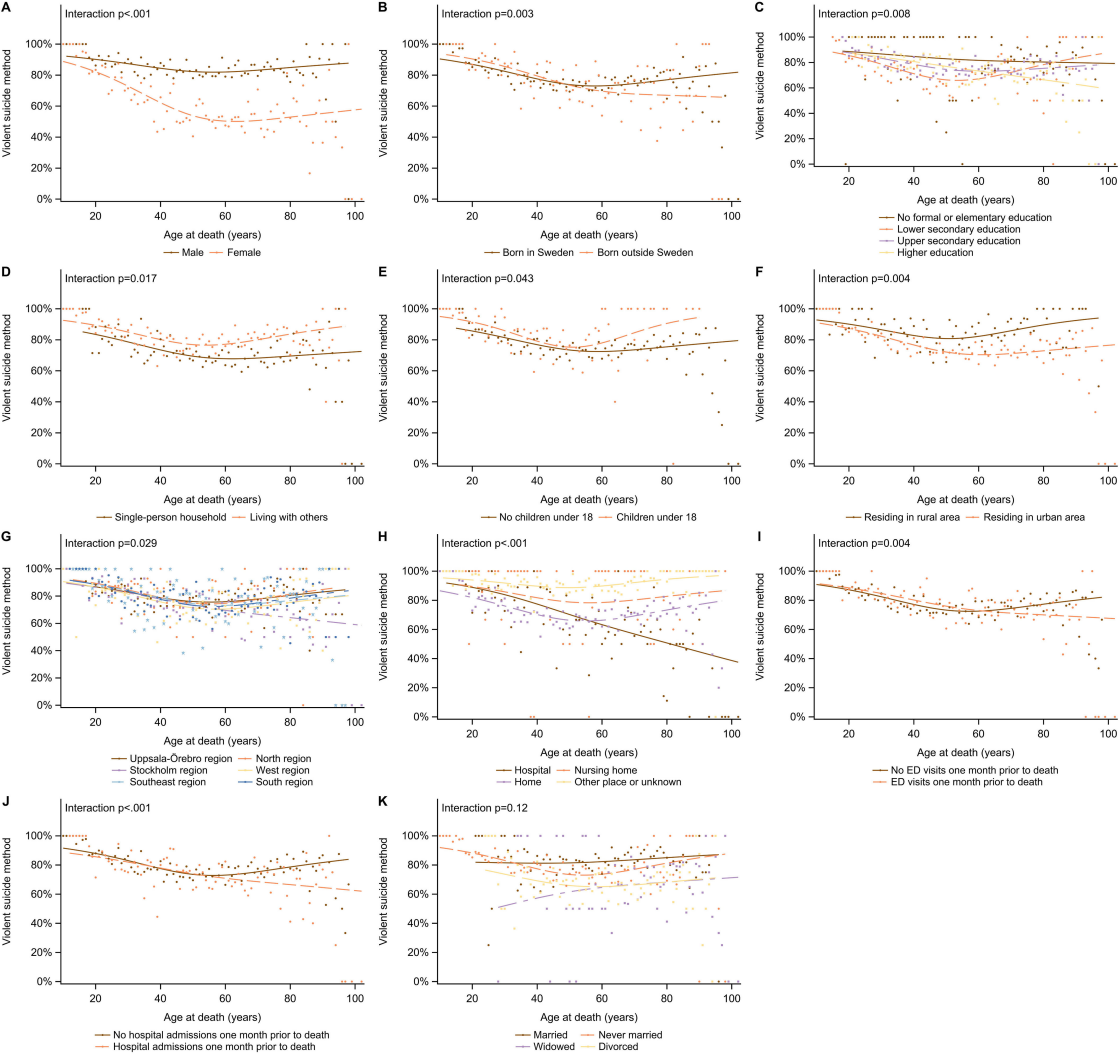
Proportion of suicides involving violent methods across subgroups defined by sex **(A)**, region of birth **(B)**, educational attainment **(C)**, living with others **(D)**, presence of children in the household **(E)**, residential area (urban/rural; **F**), healthcare region **(G)**, place of death **(H)**, emergency department visits in the month prior to death **(I)**, hospital admissions in the month prior to death **(J)**, and marital status **(K)**. Points represent observed proportions, whereas lines depict fitted mean trends estimated using logistic regression with age modelled using restricted cubic splines. P-values reflect interaction terms between age and each covariate, testing for differential age-related trends across subgroups.

Interaction analyses between age and covariates are presented in **Figure 2**, with significant interactions observed for sex (*p* <.001) (A), region of birth (*p* = .003) (B), educational attainment (*p* = .008) (C), living with others (*p* = .017) (D), presence of children in the household (*p* = .043) (E), residential area (urban/rural) (*p* = .004) (F), healthcare region (*p* = .029) (G), place of death (*p* <.001) (H), emergency department visits in the month prior to death (*p* = .004) (I), and hospital admissions during the same period (*p* <.001) (J).

Among men, the use of violent methods remained relatively stable with age, while among women it declined steadily until around age 60. After this age, foreign-born individuals were less likely than Swedish-born individuals to use violent methods, which was not seen in younger age groups. Higher educational attainment was consistently associated with lower odds of violent method use across the life stages. Age-related disparities were more pronounced among those living with others, those with children, and individuals living in rural areas. Violent methods were also less frequently used by individuals who died in hospitals, with this difference increasing with age. Similarly, older adults who had visited the emergency department or been admitted to hospital the month before death were less likely to use violent methods.

### Sex differences in factors associated with violent suicide methods

**Figure 3:**
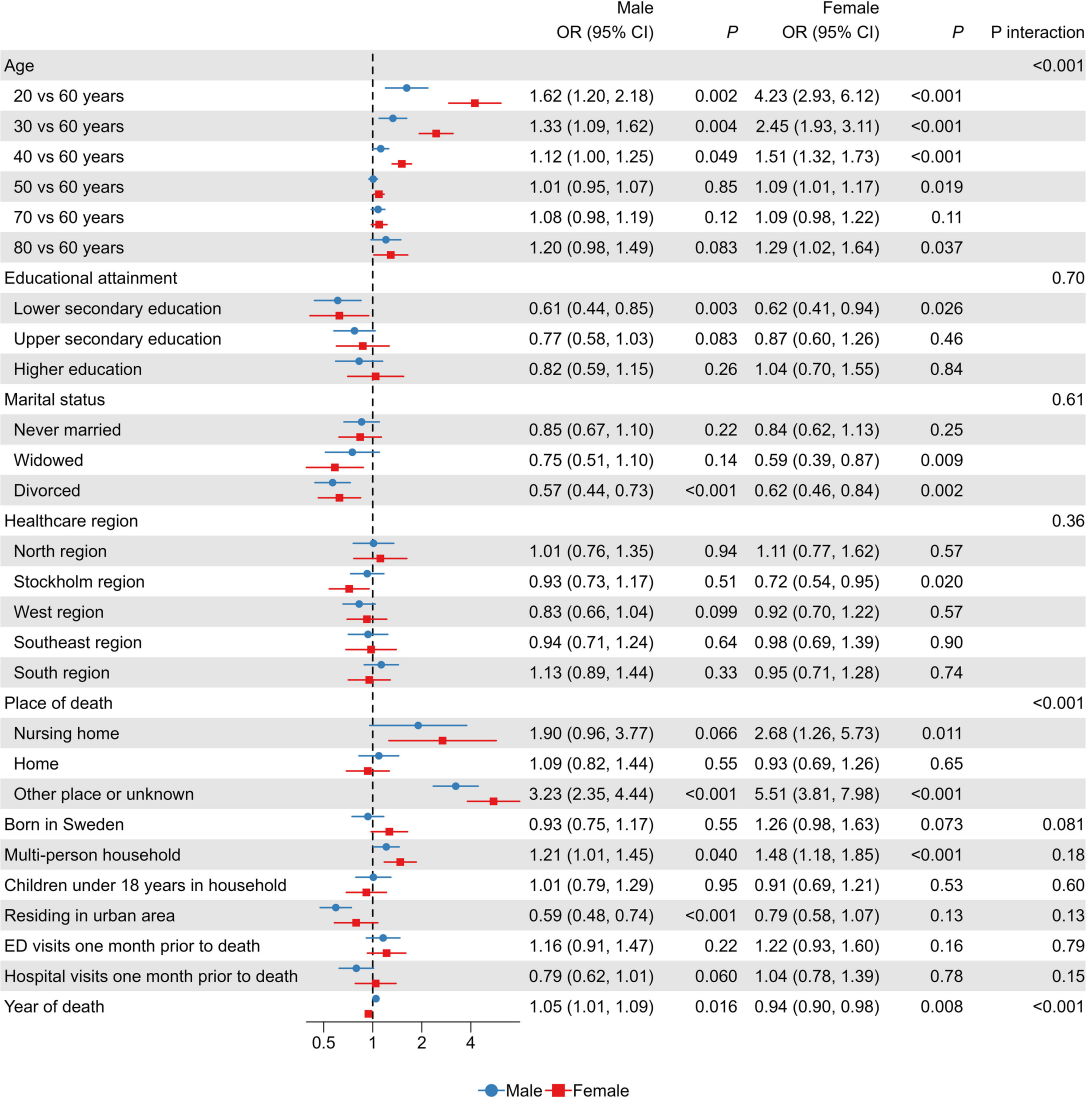
Associations between sociodemographic and clinical factors and the use of violent suicide methods, stratified by sex. Points and error bars represent odds ratios (ORs) with 95% confidence intervals (CIs), estimated using multivariable logistic regression. Results for men are shown as blue lines with circles, and for women as red lines with squares. Age was modelled using restricted cubic splines, with age 60 years set as the reference. Unspecified methods (ICD-10 X83–X84, Y87.0) were excluded. Interaction p-values test whether associations differ between men and women.

Associations between sociodemographic and clinical characteristics and the use of violent suicide methods were largely similar in men and women. In addition to age, statistically significant interactions were observed for place of death (*p* <.001) and calendar year of death (*p* <.001) (**Figure 3**). Women showed stronger associations with violent methods when the death occurred in nursing homes or in other or unknown locations. For calendar year, men showed an increasing use of violent methods over time, whereas women showed a corresponding decrease. No statistically significant sex interactions were found for educational attainment, marital status, household composition, healthcare region, or other covariates.

### *Post-hoc* analysis of hanging, poisoning, firearm, and other suicide methods

**Figure 4:**
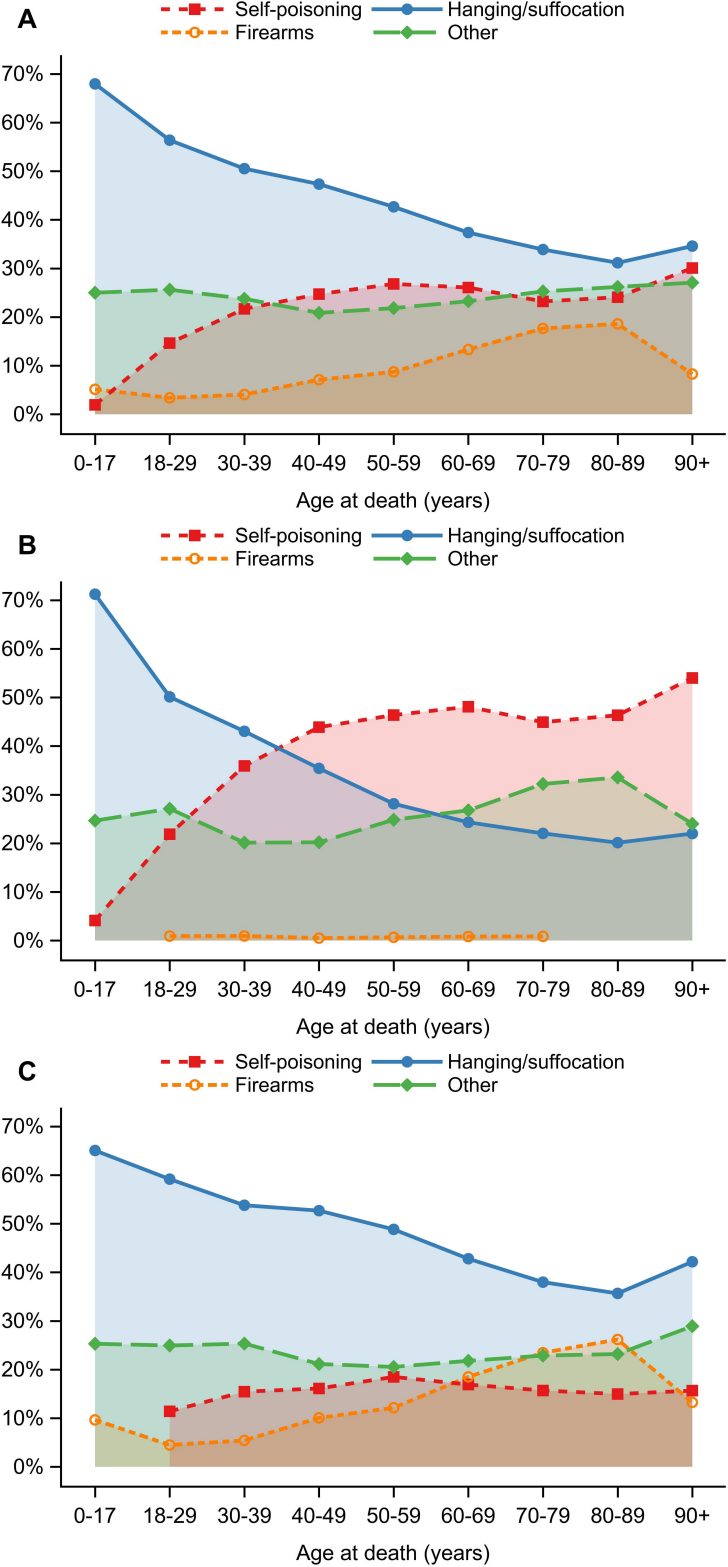
Distribution of suicide methods by age at death and sex. Line graphs show the proportion of deaths classified as hanging or suffocation, poisoning, firearms, and other methods (including drowning, cutting, jumping, and motor vehicle crashes). Panel **(A)** shows the full cohort, panel **(B)** females, and panel **(C)** males. Points represent estimated proportions within each age category and are connected by lines to illustrate age-related trends.

In a *post-hoc* analysis, we evaluated the distribution of hanging/suffocation, poisoning, firearms, and other suicide methods by age and sex (**Figure 4**). There were statistically significant differences by age and by sex (both *p* <.001), as well as a significant age–sex interaction, demonstrating that age-related variation in method choice differed between the sexes across the life course (*p* <.001). Overall, hanging/suffocation declined steadily with age for both men and women, poisoning increased into midlife (driven largely by women), and firearm suicides rose sharply in older ages (driven almost entirely by men). The “other” methods category (including drowning, cutting, jumping, and crashing of a motor vehicle) remained relatively stable across age and sex, with a modest increase among women at older ages.

## Discussion

This national population-based register study examined the use of violent suicide methods across life stages and their associations with sociodemographic characteristics, healthcare utilization, and place of death. Violent methods accounted for most suicides (77%), but the association between age and method choice varied significantly across population subgroups.

Sex emerged as the strongest predictor of suicide method. In men, the rate of violent suicide methods remained stable across the lifespan, whereas in women, it steadily declined until around age 60. This pattern is likely influenced by several factors, also observed in previous studies, whereby the use of less violent methods (e.g. self-poisoning) is higher among younger and middle-aged women, while among older women, there is a plateau or slight increase in violent methods after 60, which may be due to increased use of methods such as drowning and submersion, as well as firearms and hanging/suffocation (23, 24). Method choice in suicide is influenced by multiple factors, including availability, accessibility, and social acceptability, rather than solely violence or lethality. Women’s method preferences appear more dynamic across their life course, likely influenced by changing life circumstances, social roles, and healthcare engagement (5, 9).

We also found that foreign-born individuals over age 60 were less likely to use violent methods than their Swedish-born counterparts – a pattern not observed in younger age groups. Previous research has also shown that migrants in general have a lower risk of suicide than Swedish-born individuals (25). Many recent migrants to Sweden originate from Muslim-majority countries, where suicide rates tend to be lower and suicide patterns often reflect norms from the countries of origin (26). Despite this, a recent Swedish register study showed that individuals aged 75 and above and who were born in non-Nordic countries had a greater risk of suicide death than their Swedish-born counterparts (27). Regarding the use of violent methods among older migrants in the current study, several interrelated factors may be at work. Cultural norms play a significant role; in many non-Western societies, violent means of suicide are heavily stigmatized, particularly among older adults, who are often regarded as moral exemplars within their communities (28). Non-violent methods, such as medication overdose, may be viewed as more discrete and less disruptive to family and community, aligning with cultural values that emphasize dignity and discretion in death (29). Structural factors, including socioeconomic conditions, residential circumstances, and unfamiliarity with accessing certain violent means in Sweden, may further restrict the use of such methods in this group.

Educational attainment also showed a significant interaction with age in terms of suicide methods. Individuals with higher education were consistently less likely to use violent methods across their lifespan. Higher educational attainment may reflect a greater awareness of different suicide methods, and people with higher education probably have more access to health care and therefore greater availability of prescription medications that can be employed in non-violent suicides. Conversely, lower educational attainment may reflect underlying neurocognitive and behavioral factors, such as impaired decision-making or reduced risk appraisal, that have been associated with using violent means of suicide (30).

Older adults who had recently visited the emergency department or been admitted to hospital were less likely to use violent means. These contacts with acute care services might constitute critical windows for prevention, as patients are directly accessible for suicide risk assessment, treatment for physical and mental disorders, psychoeducation, and safety planning (31). The finding may also suggest that medical care or close monitoring in clinical settings mitigates the use of violent methods, potentially due to physical health limitations that reduce the feasibility of certain acts. Prior research supports this interpretation, showing that physical illness increases the likelihood of self-poisoning among older women, and that self-poisoning is the predominant method among hospital-based suicides, especially in later life (32; 23).

Our study highlights the importance of considering how vulnerability to violent suicide methods varies across the life course. Our data revealed a non-linear relationship with age, where the use of violent methods was most common in early adulthood, declining through midlife, and rising again in later life, which confirms patterns identified in earlier research (33). This pattern likely reflects a combination of developmental and contextual factors, including both biological transitions, such as adolescence and age-related frailty, as well as broader social and cultural dynamics that shape method choice and intent. Among younger individuals, method choice may be driven by impulsivity, social contagion, and exposure to media portrayals of suicide (34). In contrast, the increase in violent methods employed later in life may reflect greater determination combined with physical frailty that increases lethality. For older men in particular, increased access to firearms may contribute. Additionally, retirement-related losses, such as diminished social roles, status, or declining physical health may further shape method choice in this age group.

### Implications

Older adults’ increased lethality in suicide attempts due to social isolation and reduced physical resilience demands targeted prevention strategies (35). Suicide prevention needs differ significantly by age; youth crises might be more impulsive, whereas older adult crises typically involve chronic stressors, necessitating holistic treatments (36). A life-stage perspective may help identify prevention opportunities and risk pathways, emphasizing multifaceted interventions for older adults (37). Healthcare settings present critical opportunities for intervention, especially through screening for method availability as part of patient safety measures (38). Collaborative care models integrating mental health treatment within primary care effectively reduce suicidal ideation among older adults (39). Interventions targeting social support, mental health access, and pain management could significantly benefit older adults. Interventions restricting access to violent methods, notably firearms, have shown to be some of the most promising universal interventions (40).

The consistent use of violent suicide methods among men underscores the need for targeted prevention, especially among younger and older adults. Men have been shown to greatly benefit from informal support from trusted individuals. As outlined in the review by Struszczyk et al. (41), men highlighted strategies that fostered social connections. These strategies promoted help-seeking as compatible with masculine identity and taught emotion regulation techniques. Furthermore, community-wide initiatives that promote open dialogue on mental health can contribute to the dismantling of the stereotype of male stoicism. This assertion is supported by a comprehensive systematic review conducted by Sørensen et al. (42). Symptoms of depression in men are rarely discussed in the context of violent suicide or suicide attempts. For younger men, interventions might include psychoeducational programs that challenge harmful gender norms, promote emotional literacy, and offer alternative coping mechanisms (43).

### Methodological considerations

One limitation of this study is its reliance on national register data, which constrains measurement of psychological and social determinants of suicide method. A second limitation is the lack of granular diagnostic information—particularly regarding psychiatric disorders and severe physical health problems—which are well-established risk factors for suicide. To address this, we included healthcare-utilization measures; however, these captured only contacts in the month preceding death and may not reflect longer-term morbidity or service engagement. For example, Ahmedani et al. (44) used a one-year window, so our one-month measure understates healthcare contact. Besides the underlying cause of death variable, the lack of detailed diagnostic information not only limits our understanding of method choice but also obscures the relationship between mental health disorders and suicide risk across different life stages. Although the underlying cause of death variable is recommended by the WHO as it captures the initiating disease or event leading directly to death, depending solely on this variable excludes cases where self-harm was considered a contributing cause rather than the primary cause of death. Furthermore, despite the comprehensive data drawn from Sweden, findings may have limited generalizability to other countries with different cultural contexts or healthcare systems, particularly regarding firearm accessibility (45, 46).

## Conclusions

This study adds to the existing literature by identifying important age- and subgroup-specific patterns in the use of violent suicide methods. We observed a steady decline of violent methods among women until age 60, as well as a lower prevalence among older migrants. These findings highlight the importance of adopting a lifespan perspective to better understand suicide risk and method selection. They also emphasize the need for targeted, context-sensitive prevention strategies that address the unique challenges individuals face at different stages of life.

## Data Availability

This study used de-identified individual-level data from Swedish healthcare registers that were not publicly available according to Swedish legislation. The data can be obtained from the respective Swedish register holders on the basis of ethical approval for the research in question and subject to relevant legislative processes and data protection. For more information, contact the corresponding author.

## References

[1] World Health Organization . Suicide worldwide in 2019: global health estimates. Geneva, Switzerland: Mental Health and Substance Use (2021). p. 35.

[2] Castelpietra G Knudsen AKS Agardh EE Armocida B Beghi M Iburg KM . The burden of mental disorders, substance use disorders and self-harm among young people in Europe 1990–2019: findings from the global burden of disease study 2019. Lancet Regional Health - Europe. (2022) 16:100341. doi: 10.1016/j.lanepe.2022.100341, PMID: 35392452 PMC8980870

[3] The Public Health Agency of Sweden . Suicide and suicide prevention in Sweden (2023). Available online at: https://www.folkhalsomyndigheten.se/the-public-health-agency-of-Sweden/living-conditions-and-lifestyle/suicide-prevention/ (Accessed August 28, 2023).

[4] Beckman K Mittendorfer-Rutz E Waern M Larsson H Runeson B Dahlin M . Method of self-harm in adolescents and young adults and risk of subsequent suicide. J Child Psychol Psychiatry. (2018) 59:948–56. doi: 10.1111/jcpp.12883, PMID: 29504652

[5] Cai Z Junus A Chang Q Yip PSF . The lethality of suicide methods: A systematic review and meta-analysis. J Affect Disord. (2022) 300:121–9. doi: 10.1016/j.jad.2021.12.054, PMID: 34953923

[6] Bond AE Bandel SL Rodriguez TR Anestis JC Anestis MD . Mental health treatment seeking and history of suicidal thoughts among suicide decedents by mechanism 2003-2018. JAMA Network Open. (2022) 5:e222101. doi: 10.1001/jamanetworkopen.2022.2101, PMID: 35285919 PMC9907334

[7] Varnik A Kolves K van der Feltz-Cornelis CM Marusic A Oskarsson H Palmer A . Suicide methods in Europe: a gender-specific analysis of countries participating in the ‘European Alliance Against Depression’. J Epidemiol Community Health. (2008) 62:545–51. doi: 10.1136/jech.2007.065391, PMID: 18477754 PMC2569832

[8] De Silva DA Diduk-Smith RM . Comparison of suicides among younger and older adolescents in virginia 2008–2017*. Arch Suicide Res. (2022) 26:1958–65. doi: 10.1080/13811118.2021.1965929, PMID: 34425060

[9] Séguin M Beauchamp G Notredame C-É . Adversity over the life course: A comparison between women and men who died by suicide. Front Psychiatry. (2021) 12:682637. doi: 10.3389/fpsyt.2021.682637, PMID: 34447322 PMC8382958

[10] Hempstead K Nguyen T David-Rus R Jacquemin B . Health problems and male firearm suicide. Suicide Life-Threatening Behav. (2013) 43:1–16. doi: 10.1111/j.1943-278X.2012.00123.x, PMID: 23126468

[11] Nilsson AM Skärsäter I Ehnvall A Beskow J Waern M . Application of an accident approach to the study of acute suicidal episodes through repeated in-depth interviews. Death Stud. (2023) 47:75–83. doi: 10.1080/07481187.2021.2021566, PMID: 35014947

[12] Karp A . Estimating global civilian-held firearms numbers. Geneva, Switzerland: nbmedia (2018).

[13] Socialstyrelsen and Polismyndigheten . Att stärka och utveckla arbetet med tillståndsprövning för skjutvapen: En gemensam delredovisning. [Strengthening and developing the work on firearm permit assessment: a joint interim report.]. Stockholm, Sweden: Socialstyrelsen (2025). p. 59.

[14] Pirkis J Dandona R Silverman M Khan M Hawton K . Preventing suicide: a public health approach to a global problem. Lancet Public Health. (2024) 9:e787–95. doi: 10.1016/S2468-2667(24)00149-X, PMID: 39265611

[15] Brooke HL Talbäck M Hörnblad J Johansson LA Ludvigsson JF Druid H . The Swedish cause of death register. Eur J Epidemiol. (2017) 32:765–73. doi: 10.1007/s10654-017-0316-1, PMID: 28983736 PMC5662659

[16] Von Elm E Altman DG Egger M Pocock SJ Gøtzsche PC Vandenbroucke JP . The strengthening the reporting of observational studies in epidemiology (STROBE) statement: guidelines for reporting observational studies. PloS Med. (2007) 4:e296. doi: 10.1136/bmj.39335.541782.AD, PMID: 17941714 PMC2020495

[17] Ludwig B Dwivedi Y . The concept of violent suicide, its underlying trait and neurobiology: A critical perspective. Eur Neuropsychopharmacol. (2018) 28:243–51. doi: 10.1016/j.euroneuro.2017.12.001, PMID: 29254658 PMC5809305

[18] Stenbacka M Jokinen J . Violent and non-violent methods of attempted and completed suicide in Swedish young men: the role of early risk factors. BMC Psychiatry. (2015) 15:196. doi: 10.1186/s12888-015-0570-2, PMID: 26271258 PMC4536779

[19] Keilp JG Wyatt G Gorlyn M Oquendo MA Burke AK John Mann J . Intact alternation performance in high lethality suicide attempters. Psychiatry Res. (2014) 219:129–36. doi: 10.1016/j.psychres.2014.04.050, PMID: 24878299 PMC4410782

[20] Sanford AM Orrell M Tolson D Abbatecola AM Arai H Bauer JM . An international definition for “Nursing home. J Am Med Directors Assoc. (2015) 16:181–4. doi: 10.1016/j.jamda.2014.12.013, PMID: 25704126

[21] Schafer JL . Multiple imputation: a primer. Stat Methods Med Res. (1999) 8:3–15. doi: 10.1177/096228029900800102, PMID: 10347857

[22] Gauthier J Wu QV Gooley TA . Cubic splines to model relationships between continuous variables and outcomes: a guide for clinicians. Bone Marrow Transplant. (2020) 55:675–80. doi: 10.1038/s41409-019-0679-x, PMID: 31576022

[23] Choi NG DiNitto DM Marti CN Kaplan MS Conwell Y . Suicide means among decedents aged 50+ Years 2005–2014: trends and associations with sociodemographic and precipitating factors. Am J Geriatric Psychiatry. (2017) 25:1404–14. doi: 10.1016/j.jagp.2017.06.001, PMID: 28689643

[24] Martínez-Alés G Pamplin JR Rutherford C Gimbrone C Kandula S Olfson M . Age, period, and cohort effects on suicide death in the United States from 1999 to 2018: moderation by sex, race, and firearm involvement. Mol Psychiatry. (2021) 26:3374–82. doi: 10.1038/s41380-021-01078-1, PMID: 33828236 PMC8670065

[25] Niederkrotenthaler T Mittendorfer-Rutz E Mehlum L Qin P Björkenstam E . Previous suicide attempt and subsequent risk of re-attempt and suicide: Are there differences in immigrant subgroups compared to Swedish-born individuals? J Affect Disord. (2020) 265:263–71. doi: 10.1016/j.jad.2020.01.013, PMID: 32090750

[26] Voracek M Loibl LM Dervic K Kapusta ND Niederkrotenthaler T Sonneck G . Consistency of immigrant suicide rates in Austria with country-of-birth suicide rates: A role for genetic risk factors for suicide? Psychiatry Res. (2009) 170:286–9. doi: 10.1016/j.psychres.2008.10.032, PMID: 19900719

[27] Hedna K Hensing G Skoog I Fastbom J Waern M . Sociodemographic and gender determinants of late-life suicide in users and non-users of antidepressants. Eur J Public Health. (2020) 30:958–64. doi: 10.1093/eurpub/ckaa114, PMID: 32653913 PMC7536256

[28] Canetto SS . Language, culture, gender, and intersectionalities in suicide theory, research, and prevention: Challenges and changes. Suicide Life-Threatening Behav. (2021) 51:1045–54. doi: 10.1111/sltb.12758, PMID: 34515352

[29] Colucci E . Culture, cultural meaning(s), and suicide. In: Suicide and culture: Understanding the context. Hogrefe Publishing, Cambridge, MA, US (2013). p. 25–46.

[30] Lübbert M Bahlmann L Sobanski T Schulz A Kastner UW Walter M . Investigating the clinical profile of suicide attempters who used a violent suicidal means. J Clin Med. (2022) 11:7170. doi: 10.3390/jcm11237170, PMID: 36498743 PMC9735514

[31] Jensen MV Gallagher K O’Driscoll M Østervang C Christiansen E Stenager E . Suicide prevention interventions in the emergency department: A scoping review. J Emergency Nurs. (2025) 51:1097–1113.e3. doi: 10.1016/j.jen.2025.05.002, PMID: 40515748

[32] Koo YW Kõlves K De Leo D . Suicide in older adults: a comparison with middle-aged adults using the Queensland Suicide Register. Int Psychogeriatrics. (2017) 29:419–30. doi: 10.1017/S1041610216001848, PMID: 27852335

[33] Motillon-Toudic C Walter M Séguin M Carrier J-D Berrouiguet S Lemey C . Social isolation and suicide risk: Literature review and perspectives. Eur Psychiatry: J Assoc Eur Psychiatrists. (2022) 65:e65. doi: 10.1192/j.eurpsy.2022.2320, PMID: 36216777 PMC9641655

[34] Martínez V Jiménez-Molina Á Gerber MM . Social contagion, violence, and suicide among adolescents. Curr Opin Psychiatry. (2023) 36:237–42. doi: 10.1097/YCO.0000000000000858, PMID: 36762666 PMC10090320

[35] Kim SH Kim HJ Oh SH Cha K . Analysis of attempted suicide episodes presenting to the emergency department: comparison of young, middle aged and older people. Int J Ment Health Syst. (2020) 14:46. doi: 10.1186/s13033-020-00378-3, PMID: 32582367 PMC7310195

[36] Hed S Berg AI Wiktorsson S Strand J Canetto SS Waern M . Older adults make sense of their suicidal behavior: a Swedish interview study. Front Psychiatry. (2024) 15:1450683. doi: 10.3389/fpsyt.2024.1450683, PMID: 39310661 PMC11413969

[37] Hawton K Pirkis J . Preventing suicide: a call to action. Lancet Public Health. (2024) 9:e825–30. doi: 10.1016/S2468-2667(24)00159-2, PMID: 39265609

[38] Rex M Brezicka T Carlström E Waern M Ali L . Coexisting service-related factors preceding suicide: a network analysis. BMJ Open. (2022) 12:e050953. doi: 10.1136/bmjopen-2021-050953, PMID: 35450889 PMC9024253

[39] Grigoroglou C van der Feltz-Cornelis C Hodkinson A Coventry PA Zghebi SS Kontopantelis E . Effectiveness of collaborative care in reducing suicidal ideation: An individual participant data meta-analysis. Gen Hosp Psychiatry. (2021) 71:27–35. doi: 10.1016/j.genhosppsych.2021.04.004, PMID: 33915444

[40] Ishimo M-C Sampasa-Kanyinga H Olibris B Chawla M Berfeld N Prince SA . Universal interventions for suicide prevention in high-income Organisation for Economic Co-operation and Development (OECD) member countries: a systematic review. Injury Prev. (2021) 27:184–93. doi: 10.1136/injuryprev-2020-043975, PMID: 33483327 PMC8005806

[41] Struszczyk S Galdas PM Tiffin PA . Men and suicide prevention: a scoping review. J Ment Health. (2019) 28:80–8. doi: 10.1080/09638237.2017.1370638, PMID: 28871841

[42] Sørensen EH Thorgaard MV Østergaard SD . Male depressive traits in relation to violent suicides or suicide attempts: A systematic review. J Affect Disord. (2020) 262:55–61. doi: 10.1016/j.jad.2019.10.054, PMID: 31707247

[43] Åhlander A Strömbäck M Sandlund J Wiklund M . Living (dys)regulated and alienated young masculinity—Young men’s embodied experiences of mental disorders and suicidality. Counselling Psychother Res. (2023) 23:893–905. doi: 10.1002/capr.12647

[44] Ahmedani BK Simon GE Stewart C Beck A Waitzfelder BE Rossom R . Health care contacts in the year before suicide death. J Gen Internal Med. (2014) 29:870–7. doi: 10.1007/s11606-014-2767-3, PMID: 24567199 PMC4026491

[45] Choi NG Marti CN Choi BY . Three leading suicide methods in the United States 2017–2019: Associations with decedents’ demographic and clinical characteristics. Front Public Health. (2022) 10:955008. doi: 10.3389/fpubh.2022.955008, PMID: 36466504 PMC9712777

[46] Rosen T Makaroun LK Conwell Y Betz M . Violence in older adults: scope, impact, challenges, and strategies for prevention. Health Affairs. (2019) 38:1630–7. doi: 10.1377/hlthaff.2019.00577, PMID: 31589527 PMC7327526

