# Supplementary material for: Violent suicide methods across life stages - a national population-based register study
Source: Front Psychiatry. 2026 Jan 20;16:1715801. doi: 10.3389/fpsyt.2025.1715801 (PMC12864512; doi:10.3389/fpsyt.2025.1715801)
Supplement: Supplementary file 1 [file Image1.pdf]

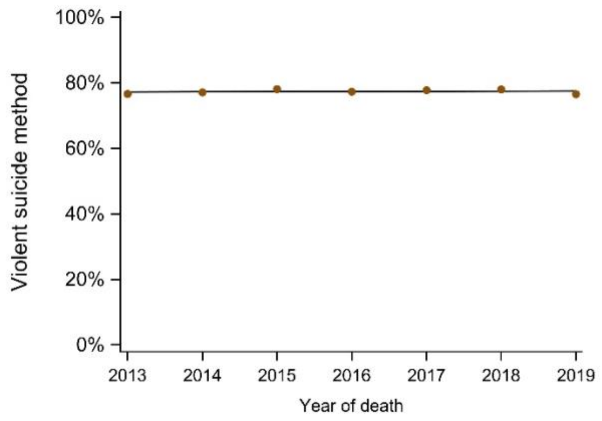

Supplementary Figure 1. Proportion of suicides involving violent methods by year of death. Points represent observed annual proportions; the line indicates the fitted mean trend estimated using logistic regression. Overall, the frequency of violent suicide methods remained stable throughout the study period.
