# Supplementary material for: Violent suicide methods across life stages - a national population-based register study
Source: Front Psychiatry. 2026 Jan 20;16:1715801. doi: 10.3389/fpsyt.2025.1715801 (PMC12864512; doi:10.3389/fpsyt.2025.1715801)
Supplement: Supplementary file 2 [file Table1.docx]

# **Supplementary Table 1.** STROBE checklist for reporting of observational studies**.**

| **Item No** | **Recommendation** | **Manuscript section** |
| --- | --- | --- |
| **1a** | Indicate the study’s design with a commonly used term in the title or the abstract | Title (“Register”), Abstract (Methods) |
| **1b** | Provide in the abstract an informative and balanced summary of what was done and what was found | Abstract – In: Methods and Results |
| **2** | Explain the scientific background and rationale for the investigation being reported | Introduction |
| **3** | State specific objectives, including any prespecified hypotheses | Introduction |
| **4** | Present key elements of study design early in the paper | Methods |
| **5** | Describe the setting, locations, and relevant dates, including periods of recruitment, exposure, follow-up, and data collection | Methods – Data sources |
| **6** | Give the eligibility criteria, and the sources and methods of selection of participants | Methods – Data sources |
| **7** | Clearly define all outcomes, exposures, predictors, potential confounders, and effect modifiers. Give diagnostic criteria, if applicable | Methods – Variables |
| **8** | For each variable of interest, give sources of data and details of methods of assessment (measurement) | Methods – Data sources, Variables |
| **9** | Describe any efforts to address potential sources of bias | Discussion – Limitations |
| **10** | Explain how the study size was arrived at | Methods – Data sources |
| **11** | Explain how quantitative variables were handled in the analyses | Methods – Statistical methods |
| **12a** | Describe all statistical methods, including those used to control for confounding | Methods – Statistical methods |
| **12b** | Describe any methods used to examine subgroups and interactions | Methods – Statistical methods |
| **12c** | Explain how missing data were addressed | Methods – Statistical methods |
| **12d** | Describe any sensitivity analyses | Methods – Statistical methods |
| **13a** | Report numbers of individuals at each stage of study | Results – Study population, |
| **13b** | Give reasons for non-participation at each stage | Not applicable – Registry data of all suicides |
| **13c** | Consider use of a flow diagram | Not included – but a comparison of the size of our study sample (N=8,325), and the total recorded deaths during the same period (599,17), are found in the Results |
| **14a** | Give characteristics of study participants | Results – Table 2, pp. 6–7 |
| **14b** | Indicate number of participants with missing data for each variable | Results – Table 2 footnotes, p. 7 |
| **15** | Report numbers of outcome events or summary measures | Results – Distribution of suicide methods, Table 3 |
| **16a** | Give unadjusted and adjusted estimates and their precision | Results – Supplementary Table 1 |
| **16b** | Report category boundaries when continuous variables were categorized | Methods – Statistical methods, e.g., age modeled with splines, |
| **16c** | Translate estimates of relative risk into absolute risk if relevant | Not applicable – Odds ratios used |
| **17** | Report other analyses done – e.g., subgroups and interactions, and sensitivity analyses | Results |
| **18** | Summarize key results with reference to study objectives | Discussion |
| **19** | Discuss limitations of the study | Discussion – Strengths and limitations |
| **20** | Give a cautious overall interpretation of results | Discussion |
| **21** | Discuss the generalizability of the study results | Discussion – Strengths and limitations |
| **22** | Give the source of funding and role of funders | Funding Statement |
